# Old Fashioned vs. Ultra-Processed-Based Current Diets: Possible Implication in the Increased Susceptibility to Type 1 Diabetes and Celiac Disease in Childhood

**DOI:** 10.3390/foods6110100

**Published:** 2017-11-15

**Authors:** Sandra V. Aguayo-Patrón, Ana M. Calderón de la Barca

**Affiliations:** Departamento de Nutrición y Metabolismo, Centro de Investigación en Alimentación y Desarrollo, A.C., Carretera a La Victoria, Km. 0.6, Hermosillo, Sonora 83304, Mexico; saguayop@gmail.com

**Keywords:** ultra-processed food products, autoimmunity risk, type 1 diabetes, celiac disease, microbiota

## Abstract

Ultra-processed foods are ready-to-heat and ready-to-eat products created to replace traditional homemade meals and dishes due to convenience and accessibility. Because of their low-fiber and high-fat and sugar composition, these foodstuffs could induce a negative impact on health. They are partially responsible for obesity and chronic non-transmissible diseases; additionally, they could impact in the prevalence of autoimmune diseases such as type 1 diabetes and celiac disease. The rationale is that the nutritional composition of ultra-processed foodstuffs can induce gut dysbiosis, promoting a pro-inflammatory response and consequently, a “leaky gut”. These factors have been associated with increased risk of autoimmunity in genetically predisposed children. In addition, food emulsifiers, commonly used in ultra-processed products could modify the gut microbiota and intestinal permeability, which could increase the risk of autoimmunity. In contrast, unprocessed and minimally processed food-based diets have shown the capacity to promote gut microbiota eubiosis, anti-inflammatory response, and epithelial integrity, through bacterial butyrate production. Thus, to decrease the susceptibility to autoimmunity, genetically predisposed children should avoid ultra-processed food products and encourage the consumption of fresh and minimally processed foods.

## 1. Introduction

Type 1 diabetes (T1D) and celiac disease (CD) are the most important autoimmune disorders in childhood; they share the genetic predisposition given by the haplotypes DQ2 and DQ8 of the human leucocyte antigen (HLA-DQ2 and -DQ8) and some environmental risk factors. In T1D, immune response to self-antigens leads to pancreatic β-cell apoptosis, lack of insulin production, and hyperglycemia [1]; while, CD is an enteropathy triggered by dietary gluten, characterized by flattened intestinal villi and impaired nutrient absorption [2]. Lately, the prevalence of T1D and CD has increased at rates that cannot be explained only by genetic predisposition [3,4,5,6], which points towards the existence of environmental factors that increase autoimmunity risk [5,7].

Factors that affect the gut microbiota balance such as feeding patterns, infections, and antibiotic treatments during the first months of life, have been associated to an increased risk of T1D and CD [8,9]. Commensal gut microbiota plays an important role in the maturation and modulation of the immune system. Misbalance in gut microbiota at an early age can induce irreversible changes in the immune response, increasing the susceptibility to allergic and autoimmune diseases [10]. Additionally, diet is a major determinant of gut microbiota profile at any age [11]. Dietary components can cause changes in microbiota composition at genus and even at phylum level, depending on the magnitude and duration of the dietary changes [12,13]. Current diets based on processed and ultra-processed foods could induce gut dysbiosis (i.e., overgrowth of opportunistic microorganisms or pathogen species). This promotes a pro-inflammatory response, an increase in gut permeability, and the susceptibility to autoimmune diseases, such as CD and T1D, in genetic predisposed children [14].

Due to its convenience and accessibility, ultra-processed foods, namely ready-to-eat or ready-to-heat products, have gained popularity in the last decades. They have substituted the “old fashioned diets”, of unprocessed and minimal processed foods, to prepare homemade meals and dishes [15]. The majority of ultra-processed products contain high energy density, fat, simple sugars, sodium, as well as several additives [16]. Their intake could induce nutritional imbalance and increase the susceptibility to autoimmunity through the modification of gut microbiota and/or other mechanisms. 

In this review, we discuss the implications of ultra-processed products-based diet and its components, in relation with gut microbiota alterations and susceptibility to autoimmunity for CD and T1D in childhood. The characteristics and beneficial potential of the minimal processed old fashioned diet and homemade meals, are also addressed.

## 2. Microbiota, Gut Health and Autoimmunity

T1D and CD share genetic predisposition, several risk factors, and bacterial markers of dysbiosis. The risk of autoimmune diseases in genetically predisposed individuals increases after alteration of gut microbiota, which leads to a pro-inflammatory response and increases gut barrier permeability [14,17]. Autoimmune diseases are associated with dysbiosis of gut microbiota; however, it remains unknown if these alterations are the cause or consequence of the disease [18]. Possibly, an aberrant gut microbiota and a “leaky gut”, induced by dietary components and/or other environmental factors, could be the main component that initiates a chain of reactions conducting to autoimmunity and preceding metabolic alterations that can reinforce dysbiosis. 

A hypothesis, proposed by Davis-Richardson and Triplett [14], suggests that a diet with high fat and gluten, and low resistant starch, induces overgrowth of the *Bacteroides* genus and reduction of butyrate producing bacteria in gut microbiota. Butyrate contributes to epithelial integrity and promotes an anti-inflammatory response [19]. The high *Bacteroides* scenario derives in the alteration of gut permeability, allowing antigens to cross to lamina propria, and increasing the susceptibility of an autoimmune response [14]. 

### 2.1. Type 1 Diabetes

The gut hypothesis of T1D etiology is explained by the common origin of the gut and pancreas, which develop from the same embryonic tissue and belong to the same intestinal immune system, in addition to the effects of dysbiosis found in both diabetes-prone mice and humans [20]. Microbiota alterations have been found in pre-clinical autoimmune stages, onset, and development of T1D. Studies have been consistent in describing that this dysbiosis is defined by an increased abundance of the genus *Bacteroides* in every stage of the disease [14,18]. 

Alkanani et al. [21], found that individuals with multiple autoantibodies for T1D had a tendency toward increased *Bacteroides* and reduced *Prevotella* abundance, compared to subjects with one autoantibody. Previously, De Goffau et al. [22] described that children with β-cell autoimmunity had low abundance of lactate and butyrate-producer bacteria. Their microbiota was characterized by lower *Bifidobacterium adolescentis* and *Bifidobacterium pseudocatenulatum*, and higher *Bacteroides* genera abundance, compared to healthy children microbiota.

The fingerprint of dysbiosis in T1D has been found even prior to seroconversion. A study in Finnish children discovered that *Bacteroides dorei* and *Bacteroides vulgatus* increased their abundance in children microbiota before developing autoimmunity, compared to controls. This change was detectable approximately eight months before the first autoantibody appearance. Thus, microbiota markers could be indicators of T1D risk in genetically susceptible children [23]. Because autoimmunity is a process that could take several years and could even be reverted [24], identifying early signs of dysbiosis offers an opportunity to delay or prevent autoimmunity by gut microbiota modulation. Improving diet quality could be the beginning of a dietary intervention aiming to modulate gut microbiota and decrease the risk of T1D development in susceptible children.

It seems that gut *Bacteroides* abundance increases in predisposed children since the first autoantibody detection until the disease onset, decreasing after treatment and metabolic control [18]. Mexican children with T1D at onset had higher abundance of *Bacteroides* than their healthy counterparts (44% vs. 12%). This abundance decreased in controlled T1D children treated for at least two years (23%), but did not reach the level of healthy children [18]. As commented previously, *Bacteroides* is associated with a pro-inflammatory immune response and an increase of para-cellular gut permeability, which could affect metabolic control of the disease or vice versa [17,18]. 

### 2.2. Celiac Disease

CD is an autoimmune enteropathy that affects the small bowel, disrupting tight junctions in the epithelia and increasing permeability as the first steps of its physiopathology [25]. Gut dysbiosis is capable to induce these conditions, and gluten peptides could be introduced to lamina propria, facilitating an immune response in genetically susceptible individuals. Additionally, epithelial damage caused by the CD immune response could provoke alterations in gut microbiota [8]. 

Studies of microbiota in CD are not as consistent as in T1D. This could probably be because, excepting young children with the classical presentation, symptoms could be milder and onset at any time in life. Still, some studies have found high abundance of the *Bacteroides* genus in the microbiota of active or treated CD patients. For example, Collado et al. [26], compared duodenal and fecal microbiota of untreated and treated pediatric CD patients and healthy controls with qPCR. *Bacteroides* and *Clostridium leptum* were more abundant in feces and biopsies of CD patients than healthy controls, regardless the disease stage. Meanwhile, *Bifidobacterium* levels were higher in healthy children than in CD patients. 

Likewise, Di Cagno et al. [27] compared microbiota of treated CD children with that of healthy controls using PCR-denaturing gradient gel electrophoresis (DGGE). They found that *Bacteroides*, *Staphylococcus*, *Salmonella*, *Shighella*, and *Klebsiella* were significantly more abundant in CD children than in healthy ones. Sánchez et al. [28] isolated *Bacteroides* clones from pediatric CD patients and healthy children, and analyzed virulence factors. *Bacteroides fragilis* was more frequent in CD patients than in controls, and the clones of this specie carrying virulence genes encoding metalloproteases were also more abundant in CD patients.

In a more recent study where DNA of the 16S ribosomal RNA was sequenced, D’Argenio et al. [29] found that Proteobacteria was the most abundant phylum in adult CD patients. At genus level *Neisseria* (from Betaproteobacteria class) was more abundant in active CD patients than in gluten-free diet controlled CD patients and in healthy controls. Moreover *Neisseria flavescens*, a potential pathogen, was the most abundant specie in active CD patients [29]. Results appear to contrast whit those found in CD children, however the technique used for microbiota analysis in the latter study is more sensitive and gives an extended panorama of the differences between groups. More detailed microbiota profile studies are still needed in pediatric CD patients.

Scenario of epithelial damage and dysbiosis generated by CD could get worse due to gluten-free diet. Frequently, gluten-free products have an imbalanced nutritional composition [30,31,32]. High consumption of sugar and fat, as in gluten-free products, is associated to gut microbiota dysbiosis [12]. Thus, it is important to review the nutritional characteristics of gluten-free products before providing them to children. Removing gluten from diet can be accomplished with an “old fashioned” diet, based on natural gluten-free, unprocessed or minimally processed foods.

## 3. Newer Is Not Always Better

Current lifestyles have influenced dietary habits from a diet based on fresh and minimal processed foods to another with high consumption of processed and ultra-processed foodstuffs [33]. Nowadays, due to the parent’s lack of time, children end up eating ultra-processed and fast foods on a regular basis [34]. 

**Figure 1:**
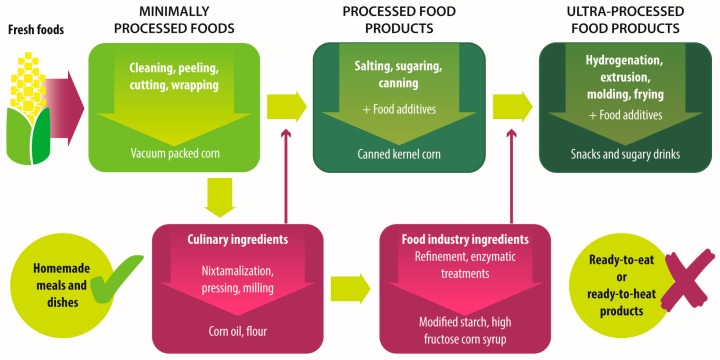
Industrial processing stages, from fresh food to ultra-processed food products.

Food processing comprises all the methods and techniques used to prepare products from fresh food and other ingredients [15]. According to the processing degree and purpose, food products can be unprocessed or minimally processed foods, processed culinary or food industry ingredients, processed foods and ultra-processed food, and drink products [15,35]. Ultra-processed foods include ready-to-eat and ready-to-heat products that replace homemade meals and dishes. They are formulated with substances derived or extracted from foods and with an extensive use of additives [15]. As a result, ultra-processed products frequently have an obesogenic nutrient profile, being less satiating and having higher glycemic loads than those of minimally processed foods [36,37]. Figure 1 shows an example of the stages and purposes of food processing, as well as the names of food products in each stage, from fresh and minimally processed to ultra-processed food products. A more extended description of food classification according to their processing degree is shown in Table 1. 

Consumption of processed and ultra-processed food products has increased in the last decades in both developed and developing countries. For instance, from 1987 to 2009, its consumption increased in Brazil from 20.3% to 32.1%. Sausages, ready meals, sweets, soft drinks, and other sugary drinks increased more than double in every socioeconomic level, with higher tendency in the lower ones [38]. According to the Pan American Health Organization, from 2000 to 2013, the sales of ultra-processed products growth from 1.5% to 68.4% among thirteen Latin American countries [35]. Similarly, in Canada there was an increase in the caloric share of ready-to-eat products from 28.7% to 61.7%, in the period of 1938 to 2011, displacing the consumption of fresh or minimally processed foods [33]. 

The disproportionate consumption of ultra-processed food products is adversely impacting general health. An ecological study made in nineteen European countries found that dietary energy from ultra-processed foods ranked from 10.2% to 50.7%, and its consumption positively correlated with the prevalence of obesity [16]. Also, in a Spanish cohort of middle-age adults, there was a positive association between ultra-processed food consumption and hypertension risk [39], which can be associated to salt content and other lifestyle behaviors.

The negative effects of ultra-processed foodstuffs on children can be even more dangerous, influenced by aggressive marketing [40]. In Brazil, processed and ultra-processed food products contributed with 19.7% and 37% of the energy intake in children younger and older than 24 months, respectively [41]. These products, in addition to the high total, saturated, and/or trans-fat, free sugars and sodium [40], have additives as emulsifiers, preservatives, colorings, and flavorings that also represent health risks. In Brazilian adolescents, ultra-processed products consumption was associated with metabolic syndrome [42] and it was a predictor of higher increase of total cholesterol and LDL cholesterol from preschool to school age [43]. Because of high glycemic load, the frequent intake of these products can induce obesity and insulin resistance in genetically predisposed children, which accelerate beta-cell apoptosis, conducing to an early T1D onset [44].

## 4. Dietary Components Shape Gut Microbiota

Diet is the main factor that influences gut microbiota composition [45]. Dietary impact has been actively studied in the last years, after the pioneering study of De Fillipo et al. [46]. They found a remarkable difference in the microbiota profile between children from a rural community in Africa and those of an urban area in Italy. African children consumed a grain-based diet with high dietary fiber intake and their microbiota was abundant in Bacteroidetes, especially in the *Prevotella* genus; meanwhile Italian children had a westernized diet (high in fat and animal protein) and Firmicutes (mainly *Faecalibacterium*) dominated their microbiota.

Subsequently, other studies found that dietary components, like fat, carbohydrates, protein, fiber, and some food additives, were associated to specific changes in the microbiota composition [12,47]. These findings are significant during infancy and childhood, because lactation regime (formula or breastfeeding), age and type of solid food introduction, and subsequent diet composition, profoundly impact microbiota establishment and maturation possibilities [11,48,49]. Surprisingly, many food products exclusively designed for infants such as baby foods, milk formulas, and ready-to-eat breakfast cereals, are in the classification of ultra-processed food products [15,35]. 

In children, an adult-like microbiota is established around 3–5 years old and its composition remains relatively stable throughout life [50], except by 30–40% of its structure, which can be modified by diet and other environmental factors [49]. Depending on dietary components, changes can be positive or unfavorable. For example, the shift to an animal-based diet (high fat and protein content) increases the abundance of bile-tolerant microorganism like *Alistipes*, *Bilophila*, and *Bacteroides* [13]. Thus, if specific diet components support particular bacterial abundance, the impact of ultra-processed food consumption on microbiota structure can be foreseen through their ingredients.

### 4.1. Complex Carbohydrates vs. Sugars

More than 70% of dietary carbohydrates in unprocessed or minimally processed diets are starches, mainly supplied by cereal grains and root vegetables. According to their structure, starches are differentially digested by gastrointestinal enzymes; the easily digested starches are absorbed in the small intestine after full digestion while the resistant starches, non-starch polysaccharides, and oligosaccharides, can reach the colon and are fermented by the microbiota [49]. In this way, consumption of resistant starches (natural or technologically made) has been considered an important dietary factor that promotes gut eubiosis and an anti-inflammatory response [14]. 

Resistant starch has been associated with colonic microbiota changes. Abundance of *Dorea*, *Ruminococcus*, and *Roseburia* spp., three butyrate producer bacteria, was increased in the colon and cecum of mice fed with resistant starch [51]. In humans, a double-blinded, crossover study in subjects with signs of metabolic syndrome, found that abundance of *Bacteroides ovatus*, *Ruminococcus lactaris*, *Eubacterium oxidoreducens*, *Bacteroides xylanisolvens*, and *Bacteroides acidifaciens* were increased after a dietary intervention with chemically modified starch. Also, proportions of butyric, propionic, valeric, isovaleric, and hexanoic acids were higher after the intervention [52]. Thus, resistant starches are dietary components with the potential to modulate the microbiota to promote a healthy gut and possibly to reduce the susceptibility to develop T1D and CD.

Non-starch polysaccharides as cellulose and other fibers are mainly found in unprocessed or minimally processed vegetables, cereals, and grains. Landmark study by De Filippo et al. [46] encountered that rural African children’s diet was high in fiber and resistant starch, due to the high intake of cereals, legumes, and vegetables. This was linked to a high abundance of *Xylanibacter*, *Prevotella*, *Butyrivibrio*, and *Treponema* in their microbiota, which can produce high levels of short chain fatty acids by fermentation of xylane, xylose, and carboxymethylcellulose. Hald et al. [53] found that a combination of arabinoxylan and naturally resistant starch-enriched diet increased the abundance of *Bifidobacterium* and production of butyrate and acetate in adults with metabolic syndrome. In addition, there were reductions in the proportions of *Bacteroides*, *Parabacteroides*, *Butyricimonas*, *Odoribacter*, and *Paraprevotella* genera, which have been associated to dysbiosis.

In contrast to the beneficial effects of resistant starch and dietary fiber, high intake of free sugars could induce dysbiosis. Sugars are extensively added to the formulation of ultra-processed foodstuffs, which are massively consumed by children; for instance in USA, these foods contribute with 89.7% of the energy intake from added sugars [54]. In an animal model, high sugar intake at early age modifies microbiota independently of obesity, reducing *Prevotella* and *Lachnospiraceae incertae sedis*, while increasing *Bacteroides*, *Alistipes*, *Lactobacillus*, *Clostridium sensu stricto*, *Bifidobacteriaceae*, and *Parasutterella* [55].

Recently, Nakayama et al. [56] found that children from an urban city in Philippines had a *Bacteroides*-dominated microbiota related to high dietary intake of free sugars, amino acids, and lipids, compared to children from a rural area whose microbiota was abundant in *Prevotella*. Children in the urban area had a higher intake of ultra-processed food products; they ate frequently fast food, more meat or meat products and confectionary (i.e., biscuit and sweetened pastry), than the rural children.

According to Schoen et al. [57], in spite of a previous notification to the mothers about the T1D risk of their children, those at-risk had lower quality diet and higher intake of added sugars than not-at-risk infants at nine and twenty-four months-old. Similar to this, Weber et al. [58] described that diet from T1D at-risk children was above the recommendation for snacks and sweet products, and below the recommendation for fruits and vegetables. In addition to altering the gut microbiota, excessive sugar intake increases insulin production, stress beta cells and increases the risk of T1D. In children with high genetic risk or islet autoimmunity, the progression to T1D has been associated to total sugar intake and sugar-sweetened beverages consumption, respectively [59]. Considering this, and that T1D and CD share genetic and environmental risk factors, to maintain an adequate dietary pattern since early infancy should be the main strategy to reduce the susceptibility to autoimmunity.

### 4.2. Unsaturated vs. Saturated Fats

Monounsaturated and polyunsaturated fatty acids can be found in unprocessed or minimally processed foods such as nuts, vegetal oils, blue fishes, and others. Their effects on the gut microbiota modulation have been demonstrated in human beings and animal models. Byerley et al. [60], added walnuts to rats’ diet and the microbiota diversity and abundance of probiotic-type bacteria such as *Lactobacillus*, *Ruminococcus,* and *Roseburia*, was increased, while *Bacteroides* and *Anaerotruncus* genus abundance was reduced. Similar results were found after supplementation of mice diet with ω-3 polyunsaturated fatty acid [61]. In a case study by Noriega et al. [62], the supplementation of a 45-year-old male with ω-3 fatty acid (600 mg/day for 14 days) increased the abundance of *Blautia*, *Coprococcus*, *Ruminococcus*, *Subdoligranulum*, and *Roseburia*. However, after a 2-week washout period, *Faecalibacterium* and *Bacteroides* increased, and the butyrate producer bacteria decreased. 

In murine models, supplementation with polyunsaturated fatty acids has proved benefits in the prevention of T1D and CD. Bi et al. [63], gave a diet enriched in ω-3 polyunsaturated fatty acids to non-obese diabetic mice, inducing an increase in the differentiation of T regulatory lymphocytes, while inflammatory cytokines such as interferon gamma, interleukin-17, interleukin-6, and tumor necrosis factor alpha decreased. This result could provide an insight at the anti-inflammatory mechanisms of ω-fatty acids. In a similar way, Bergamo et al. [64], supplemented with a conjugated linoleic acid mixture, mice with previous gliadin-induced enteropathy (model of CD). The treatment protected mice against the accumulation of oxidative stress markers and increased the number of mucin-producing cells in duodenum.

The fraction of the diet composed of ultra-processed products has at least 30% more total fat and exceeds the recommended 10% of saturated fats, compared with unprocessed and minimally processed foods [37]. Excess of total and saturated dietary fat, alters the gut microbiota and promotes a pro-inflammatory response. Mice fed a high fat diet had more gram-negative bacteria from phylum Bacteroidetes and Proteobacteria compared to those fed with a low fat diet [65]. In the same way, a diet high in fat and sucrose produced an inflammatory environment in mice’s gut, induced an overgrowth of pro-inflammatory Proteobacteria, and reduced the anti-inflammatory short chain fatty acids concentration [66].

*Bacteroides* genus abundance in gut microbiota positively correlates with trans-, saturated, animal fat, and cholesterol intake [12]. Shankar et al. [67], compared gut metabolites and microbial composition of Egyptian and US American children. They found that metabolites in American children’s gut were related to amino acids and lipids metabolism compounds (i.e., free amino acids, bile acids, taurine, and choline), meanwhile Egyptian children had higher concentrations of short chain fatty acids from polysaccharides degradation. This was related to a *Bacteroides* and a *Prevotella*-dominated microbiota in American and Egyptian children, respectively [67]. Metabolite and microbiota profiles correspond to the differences in dietary patterns between both populations, marked by a high consumption of ultra-processed products by the American children [54].

Altogether, these findings indicate that the anti-inflammatory properties and the capacity of modulate gut microbiota of unsaturated and polyunsaturated fatty acids, could reduce susceptibility to T1D and CD autoimmunity. However, more than addition of supplements to the children diet, a balanced diet with unprocessed or minimally processed foods rich in unsaturated and polyunsaturated fatty acids, should be promoted.

### 4.3. Plant vs. Animal Protein

Presently, there is concern about the excessive consumption of protein (mainly animal) in western and westernized countries [68]. As a group, ultra-processed products have 150% less protein than unprocessed foods, but fast food dishes, ready-to-heat meals, and meat products are based on animal protein accompanied of fat [37]. High abundance of *Bacteroides* correlates positively with dietary intake of animal protein, amino acids, and saturated fat mainly from meat or meat products [12]. Since the first year of life, meat consumption is associated with high *Bacteroides* abundance, as described in 6 months-old children genetically predisposed to T1D [11]. In addition, metagenomic analysis reveals that microbiota in children with westernized diet is enriched in genes encoding protein degradation pathways [56,67]. Thus high content of meat in diet, similarly to those of high fat and high sugar, could promote a pro-inflammatory state that increases the risk of allergic and autoimmune diseases [14].

Zhu et al. [69], evaluated the effect of red meat (beef and pork), white meat (chicken and fish), and non-meat (casein and soy) protein consumption in rats’ microbiota profile. Non-meat fed rats had higher abundance of *Alloprevotella*, *Roseburia*, *Prevotellaceae uncultured*, and *Bacteroides*. The white-meat group was characterized by high abundance of *Lactobacillus*; meanwhile, the red-meat group was higher in *Oscillibacter* and *Bacteroides*. There were 16 OTUs (operational taxonomic units) significantly diverse between red and white-meat groups, meaning that gut bacteria could not have big different responses to red or white meat proteins. Thereby, the contributing effect of meat to dysbiosis needs to be considered.

Infant formulas are ultra-processed products and the cow’s milk protein-containing ones have been associated to allergies and asthma in newborns [70], while those with whole casein are associated with an increased risk of T1D autoimmunity [71]. Formerly, even cow insulin and albumin were related to T1D development [72]. Cow’s milk intake incremented Bacteroidetes abundance in microbiota of rats and was related to lower butyrate and propionate concentration, whereas inflammatory markers such as tumor necrosis factor alpha were increased as compared to human milk intake [73]. Also, casein fed rats had low abundance of *Lactobacillus* in cecal microbiota [69]. This evidence, points to an adverse gut environment promoted by cow’s milk in mammalians that could increase susceptibility to allergic or immune diseases through microbiota imbalance. 

On the other hand, although gluten has a vegetal origin, it has been associated with development of several allergic and autoimmune conditions in predisposed individuals [2]. Gluten-free diet is the only treatment for CD and gluten related disorders, but nowadays, following this kind of diet has become a fashionable trend and people use it looking for a supposedly healthier lifestyle [74]. However, many of the gluten-free products have a low protein and fiber content, high total and saturated fat, and are deficient in micronutrients [30,31,32]. In addition, to replace the functional properties of gluten, in the manufacture of gluten-free products, food additives like emulsifiers and hydrocolloids are used [30,74]. Thus, due to its nutritional and processing characteristics, many gluten-free products could be classified as ultra-processed foodstuffs.

Canadian children and adolescents with CD in gluten-free diet had higher intake of fiber than their healthy counterparts, although the gluten-free diet was higher in glycemic index and load, due to the consumption of gluten-free and processed products [75]. CD children do not accomplish the recommended servings of fruit and vegetables and have a lower intake of folate than healthy control children [75]. Moreover, nutritional assessment and cardiometabolic screening has been suggested for children following a gluten-free diet due to its high content of lipids, sugars, and salt [76].

There are wide differences between a gluten-free and a wheat-based diet; therefore, after a dietary change to a gluten-free diet, the structure of gut microbiota is affected. In healthy adults, a month of gluten-free diet, reduced the abundance of beneficial bacteria such as *Bifidobacterium* and *Lactobacillus*, and increased *Enterobacteriaceae* and *Escherichia coli* counts, measured by qPCR [77]. Although these results are remarkable, qPCR does not evaluate the complete profile of microbiota and important changes in some additional bacterial genera could be missed. Another study in healthy adults, found that the gluten-free diet conduced to a lower production of short chain fatty acids as compared with a high gluten dose (30 g/day)-containing diet [78]. 

Dietary proteins should not be a problem if they are given to children according to the age recommended by the World Health Organization [79], provided by fresh or minimally processed foods. If a gluten-free diet must be followed, the overall quality of the diet should be supervised and preferably consume natural gluten-free foods rather than ultra-processed gluten-free products. 

### 4.4. Food Additives

Additives are common components of ultra-processed food products daily consumed in westernized diets. Among these, emulsifiers are extensively used in bakery and confectionary, dairy, meat, fat and oil, beverages, and candy industries. They are “surfactants” because reduce the surface tension between oil and water phases, making an emulsion more stable [80]. Due to its properties, emulsifiers affect the gut mucosal barrier integrity, increase the para-cellular and transcellular permeability, and decrease the hydrophobicity of the mucus layer [81].

Polysorbate 80 and carboxymethylcellulose are emulsifiers that promote gut inflammation [82]. The latter induced bacterial overgrowth, distended spaces between villi and increased bacterial adherence to the mucosa, in interleukin-10 gene-deficient mice [83]. Both emulsifiers induced low-grade inflammation and metabolic syndrome in wild type mice and robust colitis in predisposed mice (interleukin-10 and toll like receptor-5 deficient). This phenomenon was in a microbiota-dependent way, because germ-free mice did not develop the conditions [82]. These emulsifiers in addition to directly affect the gut barrier, stimulate the microbiota pro-inflammatory potential. High levels of bioactive flagellin were found in vitro, in a simulator of human microbiota, after an emulsifiers’ treatment [47]. 

In USA, mean dietary exposure of carboxymethylcellulose and polysorbate 80 is 27 mg/kg/day and 8 mg/kg/day, respectively. These exposure levels do not represent a safety risk according to the acceptable daily intake established by international organizations [84]. However, it should be a focus of attention that exposure to these agents is in a daily basis and since very early age, hence safety consume levels should be reviewed, especially for children at high risk of autoimmunity.

As exposed along this section, the relationship between diet and autoimmunity is mediated by gut microbiota. Dietary components have the potential to modulate the structure of gut microbiota in a positive or adverse way. Depending on diet quality, a pro-inflammatory or anti-inflammatory response could be promoted in the gut epithelia, gut permeability could be altered or not, and as consequence, susceptibility to autoimmunity could be increased or reduced. Based on the reviewed evidence, ultra-processed food products have nutrients and ingredients that enhance the susceptibility to develop autoimmunity for T1D or CD in predisposed children. Thus, if prevention is the goal, a balanced “old fashioned” diet should be provided to children since early age.

## 5. Are Old Fashioned Diets Better?

Disadvantages of ultra-processed food excessive consumption have been described along this review, but little was addressed about the benefits of a fresh and minimally processed food-based diet. Fresh and minimally processed foods are the counterpart of ultra-processed products; their content of fiber, protein, complex carbohydrates, and micronutrients is high, while sodium is low and additives are null [16]. In addition, according to the processing degree, the satiety potential of ingested foods diminishes and the glycemic load increases [36]. Table 2, presents a comparison of the characteristics of compounds and overall effects of diets composed of unprocessed foods vs. ultra-processed food products.

A diet composed of unprocessed and minimally processed foods contributes to a correct development and maintenance of gut microbiota. Eubiosis is sustained, among other factors, by an adequate balance of nutrients. Besides, an appropriate diet has the potential to correct gut dysbiosis. To prove it, Avila-Nava et al. [85], induced obesity in rats feeding them with a high fat and sucrose diet for six months, then changed it to a food combination based on a pre-hispanic Mexican diet (corn, beans, tomato, nopal, chia and pumpkin seeds). After three months of receiving this mixture, glucose intolerance decreased, lipids profile improved, and the relative abundance of *Bifidobacterium* and *Lactobacillus* increased in the gut microbiota.

The Mediterranean diet is recognized because of its multiple benefits to health. It is characterized by high intake of vegetables and fruits, legumes and whole grains, moderate consumption of red wine, and use of olive oil. In a cross-sectional study in healthy adults, Gutiérrez-Díaz et al. [86], found that individuals with high adherence to the Mediterranean diet had higher abundance of *Prevotella* and higher concentrations of fecal propionate and butyrate.

A diet high in fiber has indisputable benefits on health. The eubiosis of the gut microbiota and epithelial integrity are among those benefits. Evidence of this has been observed on individuals who follow a vegan diet. The gut microbiota profile in vegans appears to be characterized by a reduced abundance of pathobionts (i.e., opportunistic microorganisms) and a greater abundance of protective species [87]. Long term dietary fiber intake is associated to high abundance of *Prevotella* and bacteria as *Roseburia*, *Eubacterium rectale*, and *Ruminococcus bromii*, able to metabolize dietary plant polysaccharides and produce butyrate [13]. 

Finally, the attempt is not to force children at risk of autoimmunity to follow a vegan or Mediterranean diet, but to show some examples of dietary patterns that contrast with the actual trend of high ultra-processed products consumption. A balanced diet based on fresh and minimally processed food covering all the nutritional requirements, including dietary fiber and resistant starch, plus avoiding ultra-processed products, must be the adequate choice. That is, going back to the “old fashioned” or traditional homemade meals and dishes. 

## 6. Conclusions

Diet is the major factor influencing gut microbiota structure. Unbalanced dietary patterns result in dysbiosis, high fat and sugar intake increases *Bacteroides* abundance, which promotes a pro-inflammatory immune response, and increases epithelial permeability. In this way, gut microbiota dysbiosis is related to an increased risk of develop T1D or CD in genetically predisposed children. Ultra-processed food products have dysbiosis-inducing nutrient profile: high fat and sugar, and low protein and micronutrients, and are highly consumed by children. Those products also contain emulsifiers, which affect microbiota and epithelial integrity. Thus, ultra-processed food products could contribute to an increased susceptibility to T1D and CD through microbiota imbalance. Changing children dietary patterns could prevent this problem. A fresh or minimally processed food-based diet, with adequate dietary fiber, low fat and sugar intake, corrects gut dysbiosis and maintains the gut epithelia integrity. Hence, an adequate “old fashioned” diet that avoids ultra-processed products must be provided to genetically predisposed children since an early age in order to reduce the susceptibility to develop T1D or CD.

## Figures and Tables

**Table 1:** Classification of food according to its processing degree *.

Group	Definition	Processing	Examples
Unprocessed foods	Fresh foods directly obtained from plants or animals.	No industrial processing.	Fresh fruits, vegetables, meat, eggs, grains and legumes.
Minimally processed foods	Physical alteration of unprocessed foods.	Peeling, cutting, drying, pasteurization, refrigeration, freezing, vacuum packing, simple wrapping.	Chilled, frozen or dried fruits, vegetables, meat and poultry; pasteurized or powdered milk; vegetables or fruit juices without added sugar.
Processed culinary ingredients	Substances extracted from unprocessed or minimally processed foods used to prepare dishes and meals.	Pressing, refining, grinding, milling.	Salt, sugar, flour, vegetable oil, starches, butter, etc.
Processed food industry ingredients	Substances extracted from unprocessed or minimally processed foods used in the formulation of ultra-processed foods.	Hydrogenation, hydrolysis, use of enzymes and additives.	High fructose corn syrup, lactose, milk and soy proteins.
Processed foods	Products made by adding sugar, salt, oil, fats or other culinary ingredients, to minimally processed foods.	Preservation or cooking methods, non-alcoholic fermentation.	Bread, cheese, canned vegetables and legumes, fruits in syrup, salted nuts and seeds, smoked and salted meat.
Ultra-processed foods	Industrial formulations manufactured mainly from processed food industry ingredients.	Frying, deep frying, curing, extrusion, molding, extensive use of additives, such as preservatives, colorants, flavorings, non-sugar sweeteners, emulsifiers, etc.	Ready-to-heat, ready-to-eat or ready-to-drink products like carbonated drinks, sweet or savory snacks, breakfast cereals, fruit yoghurt, sausages, hams, instant soups, pre-prepared meals and dishes, infant formulas, baby food.

* Adapted from [15,35,37].

**Table 2:** Comparative characteristics of compounds and overall effects of old fashioned diet vs. ultra-processed food-based diet.

Characteristic	Old Fashioned Diet (Unprocessed or Minimally Processed Food-Based Diet)	Ultra-Processed Products-Based Diet
Fiber *	↑ Dietary fiber from vegetables, whole grains and cereals.	↓ Dietary fiber due to the refining process.
Fat *	Balance between saturated and unsaturated fats, depending on food selection.	↑ Total fat and trans fat added or generated by the processes of baking and frying.
Carbohydrates *	↑ Complexes carbohydrates and natural resistant starch from whole grains and cereals.	↑ Added sugars in sweets, confectionary and soft drinks.
Protein *	↑ Amount and quality of protein from fresh meat, eggs, fish and poultry.	↓ Quantity of protein often accompanied by added fat.
Micronutrients *	↑ Quantity of vitamins and minerals if all food groups are included in a balanced way.	↓ Concentration of vitamins and minerals due to the refining process if not fortified.
Sodium *	Sodium intake depends mainly on the added salt to foods.	↑ Amounts of sodium.
Additives *	Free of additives.	Extensive use of additives like emulsifiers, coloring, flavoring, and preservatives.
Effect on ^ç^:		
Gut microbiota ^‡^	Eubiosis with high abundance of butyrate producer bacteria.	Dysbiosis marked by *Bacteroides* and gram-negative Proteobacteria.
Bacterial Metabolites ^γ^	↑ Production of butyrate	↑ Production of acetate and other short chain fatty acids.
Immune response ^§^	Anti-inflammatory response.	Pro-inflammatory response.
Epithelia integrity ^§^	Thigh junction’s integrity due to the production of butyrate.	Altered intestinal permeability due to dysbiosis or emulsifiers’ effect.
Susceptibility to T1D or CD ^¶^	Reduced susceptibility.	Increased susceptibility.

↑: higher; ↓: lower; T1D: type 1 diabetes; CD: celiac disease; * [15,16,33,34,35,36,37,38,39,40,41,42,43,54]; ^ç^ Comparing high in fiber and resistant starch vs. high in fat, sugars and emmulsifiers diets. ^‡^ [11,12,13,14,46,49,51,52,53,56,60,61,62,63,64,65,66,67,69,85,86,87]; ^γ^ [17,51,53,56,67]; ^§^ [14,17,47,51,80,81,82,83]; ^¶^ [11,17,44,80].
